# Prospective associations between a priori dietary patterns adherence and kidney function in an elderly Mediterranean population at high cardiovascular risk

**DOI:** 10.1007/s00394-022-02838-7

**Published:** 2022-04-02

**Authors:** Cristina Valle-Hita, Andrés Díaz-López, Nerea Becerra-Tomás, Miguel A. Martínez-González, Verónica Ruiz García, Dolores Corella, Albert Goday, J. Alfredo Martínez, Ángel M. Alonso-Gómez, Julia Wärnberg, Jesús Vioque, Dora Romaguera, José López-Miranda, Ramon Estruch, Francisco J. Tinahones, José Lapetra, Luís Serra-Majem, Naomi Cano-Ibáñez, Josep A. Tur, María Rubín-García, Xavier Pintó, Miguel Delgado-Rodríguez, Pilar Matía-Martín, Josep Vidal, Sebastian Mas Fontao, Lidia Daimiel, Emilio Ros, Estefania Toledo, José V. Sorlí, C. Roca, Iztiar Abete, Anai Moreno-Rodriguez, Edelys Crespo-Oliva, Inmaculada Candela-García, Marga Morey, Antonio Garcia-Rios, Rosa Casas, Jose Carlos Fernandez-Garcia, José Manuel Santos-Lozano, Javier Diez-Espino, Carolina Ortega-Azorín, M. Comas, M. Angeles Zulet, Carolina Sorto-Sanchez, Miguel Ruiz-Canela, Montse Fitó, Jordi Salas-Salvadó, Nancy Babio

**Affiliations:** 1 Department of Biochemistry and Biotechonology, Universitat Rovira i Virgili, Human Nutrition Unit, Carrer Sant Llorenç, 21, 43201 Reus, Spain; 2 Institut ďInvestigació Sanitària Pere Virgili (IISPV), 43204 Reus, Spain; 3 University Hospital of Sant Joan de Reus, Nutrition Unit, 43201 Reus, Spain; 4 Consorcio CIBER, M.P. Fisiopatología de la Obesidad y la Nutrición (CIBERObn), Institute of Health Carlos III, 28029 Madrid, Spain; 5 Serra Hunter Fellow, Nutrition and Mental Health Research Group (NUTRISAM), Universitat Rovira i Virgili, 43201 Reus, Spain; 6 Department of Epidemiology and Biostatistics, School of Public Health, Faculty of Medicine, Imperial College London, St Mary’s Campus, Norfolk Place, London, W2 1PG UK; 7 Department of Preventive Medicine and Public Health, University of Navarra, IdiSNA, 31008 Pamplona, Spain; 8 Department of Nutrition, Harvard T.H. Chan School of Public Health, Boston, MA 02115 USA; 9 University Hospital of Tarragona Joan XXIII, 43005 Tarragona, Spain; 10 Department of Preventive Medicine, University of Valencia, 46010 Valencia, Spain; 11 Cardiovascular Risk and Nutrition Research Group (CARIN), Hospital del Mar Research Institute (IMIM), Departament de Medicina, Universitat Autònoma de Barcelona, 08003 Barcelona, Spain; 12 Department of Nutrition, Food Science and Physiology, University of Navarra, IdiSNA, 31008 Pamplona, Spain; 13 Precision Nutrition Program, CEI UAM + CSIC, IMDEA Food and Health Sciences, 28049 Madrid, Spain; 14 CEI UAM + CSIC, Nutritional Control of the Epigenome Group, IMDEA Food, 28049 Madrid, Spain; 15 Bioaraba Health Research Institute, Osakidetza Basque Health Service, Araba University Hospital, University of the Basque Country UPV/EHU, 01009 Vitoria-Gasteiz, Spain; 16 Department of Nursing, University of Málaga, Institute of Biomedical Research in Malaga (IBIMA), 29071 Málaga, Spain; 17 Instituto de Investigación Sanitaria y Biomédica de Alicante, Miguel Hernandez University (ISABIAL-UMH), 46020 Alicante, Spain; 18 CIBER de Epidemiología y Salud Pública (CIBERESP), Instituto de Salud Carlos III, 28029 Madrid, Spain; 19 Health Research Institute of the Balearic Islands (IdISBa), 07120 Palma de Mallorca, Spain; 20 Department of Internal Medicine, Maimonides Biomedical Research Institute of Cordoba (IMIBIC), Reina Sofia University Hospital, University of Cordoba, 14004 Cordoba, Spain; 21 Department of Internal Medicine, Institutd’Investigacions Biomèdiques August Pi Sunyer (IDIBAPS), Hospital Clinic, University of Barcelona, 08036 Barcelona, Spain; 22 Department of Endocrinology, Virgen de la Victoria Hospital Instituto de Investigación Biomédica de Málaga (IBIMA), University of Málaga, 29010 Málaga, Spain; 23 Department of Family Medicine, Research Unit, Distrito Sanitario Atención Primaria Sevilla, 41013 Sevilla, Spain; 24 Preventive Medicine Service, University of Las Palmas de Gran Canaria, Research Institute of Biomedical and Health Sciences (IUIBS), Centro Hospitalario Universitario Insular Materno Infantil (CHUIMI), Canarian Health Service, 35016 Las Palmas, Spain; 25 Department of Preventive Medicine, University of Granada, 18071 Granada, Spain; 26 Research Group On Community Nutrition and Oxidative Stress, University of Balearic Islands, 07122 Palma de Mallorca, Spain; 27 Institute of Biomedicine (IBIOMED), University of León, 24071 León, Spain; 28 Lipids and Vascular Risk Unit, Internal Medicine, Hospital Universitario de Bellvitge-IDIBELL, Hospitalet de Llobregat, 08907 Barcelona, Spain; 29 University of Barcelona, 08007 Barcelona, Spain; 30 Division of Preventive Medicine, Faculty of Medicine, University of Jaén, 23071 Jaén, Spain; 31 Department of Endocrinology and Nutrition, Instituto de Investigación Sanitaria Hospital Clínico San Carlos (IdISSC), 28040 Madrid, Spain; 32 Departament of Endocrinology, IDIBAPS, Hospital Clínic, University of Barcelona, 08036 Barcelona, Spain; 33 CIBER Diabetes y Enfermedades Metabólicas (CIBERDEM), Instituto de Salud Carlos III (ISCIII), 28029 Madrid, Spain; 34 Department of Endocrinology and Nutrition, University Hospital Fundación Jimenez Díaz, Instituto de Investigaciones Biomédicas IISFJD, 28040 Madrid, Spain; 35 Department of Endocrinology and Nutrition, Lipid Clinic, Hospital Clínic, Institut d’Investigacions Biomèdiques August Pi Sunyer (IDIBAPS), 08036 Barcelona, Spain; 36 Centro de Salud Santa Pola, 03130 Alicante, Spain; 37 Atención Primaria, Servicio Navarro de Salud, Osasunbidea, Pamplona, Spain

**Keywords:** Dietary patterns, Mediterranean diet, DASH diet, Protein diet score, Kidney function, Glomerular filtration rate

## Abstract

**Purpose:**

To assess the association between three different a priori dietary patterns adherence (17-item energy reduced-Mediterranean Diet (MedDiet), Trichopoulou-MedDiet and Dietary Approach to Stop Hypertension (DASH)), as well as the Protein Diet Score and kidney function decline after one year of follow-up in elderly individuals with overweight/obesity and metabolic syndrome (MetS).

**Methods:**

We prospectively analyzed 5675 participants (55–75 years) from the PREDIMED-Plus study. At baseline and at one year, we evaluated the creatinine-based estimated glomerular filtration rate (eGFR) and food-frequency questionnaires-derived dietary scores. Associations between four categories (decrease/maintenance and tertiles of increase) of each dietary pattern and changes in eGFR (ml/min/1.73m^2^) or ≥ 10% eGFR decline were assessed by fitting multivariable linear or logistic regression models, as appropriate.

**Results:**

Participants in the highest tertile of increase in 17-item erMedDiet Score showed higher upward changes in eGFR (*β*: 1.87 ml/min/1.73m^2^; 95% CI: 1.00–2.73) and had lower odds of ≥ 10% eGFR decline (OR: 0.62; 95% CI: 0.47–0.82) compared to individuals in the decrease/maintenance category, while Trichopoulou-MedDiet and DASH Scores were not associated with any renal outcomes. Those in the highest tertile of increase in Protein Diet Score had greater downward changes in eGFR (*β*: − 0.87 ml/min/1.73m^2^; 95% CI: − 1.73 to − 0.01) and 32% higher odds of eGFR decline (OR: 1.32; 95% CI: 1.00–1.75).

**Conclusions:**

Among elderly individuals with overweight/obesity and MetS, only higher upward change in the 17-item erMedDiet score adherence was associated with better kidney function after one year. However, increasing Protein Diet Score appeared to have an adverse impact on kidney health. Trial Registration Number: ISRCTN89898870 (Data of registration: 2014).

**Supplementary Information:**

The online version contains supplementary material available at 10.1007/s00394-022-02838-7.

## Introduction

Chronic Kidney Disease (CKD) is an increasing global public health problem, which affects about 9.1% of the worldwide population and 35% of those over 70 years [1]. CKD is characterized by abnormalities in kidney structure and a decline in its function [2], often accompanied by several comorbidities, decreased quality of life and premature mortality [1–3]. In fact, this heterogeneous condition is accelerated at older ages when comorbidities such as obesity, diabetes, hypertension and/or cardiovascular disease are present [1, 4]. As this disease involves a huge health and economic burden, preserving renal function, especially in old people, it is essential to ensure the well-being and reduce adverse health outcomes [1]. Accordingly, effective strategies to deal with the spread of CKD and its harmful consequences are urgently needed.

Among the lifestyle risk factors of CKD, diet may play an important role as potential modulator of kidney function decline and CKD progression [5]. However, most of the investigations have been predominantly focused on single nutrients [6, 7] or food groups [5, 8] instead of dietary patterns, hence it is likely to not exhibit the synergistic effect between its dietary components. Thus, considering that meals are composed by a combination of foods and nutrients, analysis of diet as a whole could be a more all-inclusive approach not only to assess dietary exposure but also to examine its relationship with kidney health [3, 9, 10].

In this regard, results of a recent meta-analysis of prospective studies reported that a healthy dietary pattern characterized by a high consumption of plant-based food was associated with reduced incidence of CKD or albuminuria, but not with glomerular filtration rate (GFR) decline [11]. When studies are focused on particular dietary patterns such as the Mediterranean diet (MedDiet) or the Dietary Approach to Stop Hypertension (DASH), which are the most commonly investigated a priori dietary scores in the context of CKD [3, 12], there are inconsistent results. Whereas some studies reported a decrease in GFR decline [13], microalbuminuria or lower CKD risk [14], others failed to demonstrate any relationship between these dietary patterns and kidney outcomes [15, 16]. It is noteworthy that most of these epidemiologic studies were conducted in healthy young or middle-aged individuals, instead of high-risk participants such as elders or people with cardiometabolic comorbidities.

Furthermore, MedDiet and DASH diet are characterized by a high plant protein content and, even though protein intake has been previously associated with kidney function deterioration [17], this potential harmful effect could depend on the protein source. Accordingly, it may be of interest to investigate the association between not only the quantity but also the quality of dietary protein and renal function using specific tools as the recently developed Protein Diet Score [18].

Consequently, in view of the above, further research is required to clarify whether specific healthy dietary patterns could preserve kidney function and even prevent its decline in elderly population with underlying comorbid conditions. Therefore, the main aim of the present study was to prospectively evaluate the association between changes towards an increase in the adherence to three a priori dietary patterns (17-item erMedDiet Score, Trichopoulou-MedDiet and DASH) and kidney function decline after 1 year of follow-up in a large Spanish cohort of middle-aged individuals with overweight/obesity and metabolic syndrome (MetS). Secondarily, we evaluated the association between changes in the Protein Diet Score and kidney function.

## Material and methods

### Study design and participants

In the present study, data was analyzed using an observational prospective design conducted within the framework of the Prevención con Dieta Mediterránea (PREDIMED)-Plus trial, which included 6874 older adults enrolled between 2013 and 2016 by 23 Spanish centers working in collaboration with primary care clinics. The study design and protocol have been described in detail elsewhere [19], and are available at http://www.predimedplus.com. In brief, the PREDIMED-Plus study is an ongoing, 8-year, parallel-group, randomized and controlled clinical trial which combines dietary and physical activity intervention with behavioral support for primary cardiovascular prevention. Eligible participants were men (55–75 years) and women (60–75 years) with overweight or obesity (BMI ≥ 27 kg/m^2^ and < 40 kg/m^2^) and free from cardiovascular disease who satisfied at least 3 criteria for the MetS definition [20]. More specific details of inclusion/exclusion criteria have been previously reported [19]. All participants provided written informed consent and the Institutional Review Boards of each participating center approved the final study protocol and procedures, which followed the standards of the Declaration of Helsinki. The trial was registered in 2014 at the International Standard Randomized Controlled Trial registry (https://www.isrctn.com/ISRCTN89898870).

For the current analysis, we excluded 777 participants who did not complete food frequency questionnaires (FFQ) at baseline and after a 1 year of follow-up; 160 participants with extreme total energy intake (women < 500 and > 3500 kcal/day, and men < 800 and > 4000 kcal/day) [21]; and 262 participants with missing creatinine data at baseline and after 1 year of follow-up. Consequently, the final sample for the present study was 5675 participants. Supplementary table S3 depicts the baseline characteristics by included and excluded participants.

### Dietary assessment

Dietary intake was evaluated at baseline and yearly during the follow-up using a 143-item semi-quantitative FFQ [22]. Trained dieticians asked the participants about their frequency consumption of each specific item during the preceding year in a face-to-face interview. There were nine possible answers ranging from never or almost never to more than six times per day. Each item answer was collected in standard portion sizes and then transformed to g/day. Total daily intake of energy, nutrients and food groups were estimated using Spanish food composition tables [23, 24].

Moreover, in order to assess the degree of adherence to an energy reduced-MedDiet of each participant, a recently validated 17-item test (17-items erMedDiet Score) [25] was filled in every visit. This questionnaire specifically asked about the frequency consumption of extra virgin olive oil, other fats (butter, margarine, or cream), fruits and fruit juices, vegetables, meat, legumes, fish, nuts, pastries, “sofrito”, non-caloric artificial sweeteners, refined grains, wholegrains, and wine. Each item of the questionnaire is scored with 1 or 0 points, when the criterion is met or not met respectively, ranging the overall score from 0 to 17 points, with 0 meaning no adherence and 17 meaning maximum adherence. Hence, this pre-defined questionnaire has the remarkable characteristic of being based on the scientific knowledge on what is a health level of intake and not on the study population distribution, unlike other indices that are calculated based on specific cut-off points (mean or quintiles) of the population included for each item of the score.

We also constructed the Trichopoulou-MedDiet Score and the DASH Score following previous detailed description in the scientific literature [26, 27]. Briefly, Trichopoulou-MedDiet Score, one of the most used MedDiet scores in nutritional epidemiological studies, is comprised of nine components and a sex-specific cutoff point, based on the median of each item consumption (g/day), is established. Components which consumption is highly recommended were 1 point scored when their consumption was equal to or greater than the median (vegetables, fruits and nuts, legumes, cereals and potatoes, fish and others, monounsaturated: saturated ratio) and zero otherwise. Non favourable components were scored with 0 when their intake was equal to or greater than the median (meat and meat products, dairy) and one otherwise. With regard to alcohol, it has been recommended a moderate consumption; therefore, the consumption of 10–50 g/day in men and 5–25 g/day in women was scored with 1 point. Alcohol consumption above or below these limits was assigned a score of 0. The total final score ranged from 0 to 9 points. For DASH Score, study population was classified into quintiles according to each score item (g/day). A progressive score from 1 to 5 was given to each quintile in case of highly recommended foods (fruits, vegetables, legumes and nuts, low-fat dairy, and whole grains). Nevertheless, an inverse score was assigned to components quintiles whose consumption is not recommended (sodium, red and processed meats, and sweetened-sugar beverages). Thus, the total score after summing up every single component ranged from 8 to 40 for DASH score.

We also included the pre-defined Protein Diet Score in our analyses [18]. The study population was divided in 11 strata according to total protein intake (E%) and in another 11 strata according to plant to animal protein ratio. Subjects in the highest stratum received 10 points and those in the lowest stratum received 0 points. Therefore, the overall score could range from 0 to 20 points, with 0 meaning the lowest total protein intake (E%) and lowest plant to animal protein ratio, and 20 meaning the highest total protein intake (E%) and the highest plant to animal protein ratio. Each component of the score was also considered separately.

### Ascertainment of renal function

For the present study, we considered as main outcomes of interest, absolute changes in eGFR and ≥ 10% eGFR decline after 1 year of follow-up. For this purpose, eGFR was estimated indirectly from serum creatinine (SCr) at baseline and after a 1 year of follow-up using the Chronic Kidney Disease Epidemiology Collaboration equation for Caucasian individuals (CKD-EPI) [28]. Fasting blood samples were collected and SCr levels were determined by the enzymatic creatinine assay method (coefficient of variation < 4%). Decline in eGFR (≥ 10%) was estimated using the following formula: (one-year eGFR− baseline eGFR)/(baseline eGFR)*100. Participants were categorized in those with *a* ≥ 10% eGFR decline and those with < 10% eGFR decline [29].

### Assessment of other covariates

At baseline and after 1 year of follow-up, socio-demographic (age, sex, educational level) and lifestyle (physical activity, smoking habits) information, medication use and history of disease were collected by PREDIMED-Plus-trained staff through several questionnaires or reviewing medical records. Anthropometric measurements as weight, height or waist circumference were assessed following the study protocol. Body Mass Index (BMI) was calculated by dividing the weight (in kg) by the square of the height (in meters). For weight change after 1 year of follow-up, we constructed a new covariate by subtracting weight at 1 year minus weight at baseline. Resting blood pressure was measured in triplicate by an automated digital device (Omron-HEM297705C).

### Statistical analyses

Data were analyzed using the available PREDIMED-Plus database updated to December 2020. Participants were categorized in four categories according to changes in dietary pattern scores after 1 year of follow-up: decrease or maintenance of changes and tertiles of increasing changes. Baseline characteristics of the study population were compared among categories of dietary patterns changes by using one-way ANOVA and Chi-square, as appropriate. Values were presented as percentages and numbers for categorical variables and means ± standard deviations for continuous variables.

Multivariable linear regression models were fitted to evaluate associations between categories of changes in dietary patterns and changes in eGFR (ml/min/1.73m^2^); results were expressed as β-coefficients and their 95% confidence interval (CI). Besides, logistic regression models were used to assess the association between categories of changes in dietary patterns and eGFR decline (≥ 10%); results were expressed as odds ratios (OR) and their 95% CI. In both regression models, the first category (decrease or maintenance of changes) was used as reference. Changes in dietary pattern exposures were also modeled as continuous per 1-point increase. All regression models were adjusted for several potential confounders: Model 1 was adjusted for sex and age; model 2 was additionally adjusted for BMI (kg/m^2^), smoking habits (never, current or former smoker), educational level (primary, secondary education or graduate), leisure time physical activity (METS/min/week), diabetes prevalence (yes/no), hypertension prevalence (yes/no), hypercholesterolemia prevalence (yes/no), center (categorized into quartiles by number of participants), intervention group and energy intake (kcal/day); and model 3, additionally adjusted for each baseline dietary pattern score and 1-year changes in body weight. All linear regression models were further adjusted for baseline eGFR (ml/min/1.73m^2^).

We used the robust variance estimators to account for intra-cluster correlations in all regression models. The *p* for linear trend was calculated assigning the median value of each category and modelling it as a continuous variable.

We also evaluated whether the associations observed between categories of changes in dietary pattern scores and changes in eGFR could be modified by sex, age, diabetes, and intervention group. Interaction was tested with the likelihood ratio tests, which involved comparing models with and without cross-product terms.

An additional analysis was included to assess the importance of each individual item of the 17-item erMedDiet Score on changes in eGFR and ≥ 10% decline in eGFR. Following a previous described method [30], each item was consecutively removed one at a time from the total score. The exclusion of each item reduced the total score to 16 items. Therefore, to assure comparability with the original score, which could have a maximum punctuation of 17, we multiplied the estimated β-coefficients and the logarithm of the estimated odds ratios by 16/17. The latest was back-transformed to the original scale through exponentiation.

Statistical analyses were performed with Stata/SE software, version 14.0 (StataCorp, College Station, TX). All tests were considered significant at a 2-tailed *p* value < 0.05.

## Results

Baseline characteristics of the study population according to 1-year changes categories (decrease/maintenance *vs*. tertile 3) of 17-item erMedDiet, Trichopoulou-MedDiet and DASH dietary pattern scores are presented in Table 1. Participants located in the highest tertile of increase in the 17-item erMedDiet score were less likely to be older, women and physically active, had less prevalence of diabetes, had more energy intake and consumed less proteins. Participants with higher increase in the Trichopoulou-MedDiet score adherence were more likely to exercise, had higher hypertension prevalence, less energy intake and consumed more proteins. Those individuals with higher increase in the DASH score were younger and mainly men. They had a higher energy intake and consumed less protein. Furthermore, differences in smoking status and education level were also observed. Regarding baseline scores of each dietary pattern, participants who most changed their adherence to the corresponding dietary pattern after one year of follow-up showed lower points at the beginning of the study. Baseline characteristics of the population under study according to the four categories of changes of each dietary pattern and Protein Diet Score are displayed in Supplementary Tables 1 and 2.

**Table 1:** Baseline characteristics according to categories of changes in the Mediterranean diet (17-item erMedDiet score and Trichopoulou) and the Dietary Approaches to Stop Hypertension (DASH) scores after 1 year of follow-up in the PREDIMED-PLUS cohort

	Δ Mediterranean diet (17-item erMedDiet score)		*p* value	Δ Mediterranean diet (Trichopoulou)		*p* value	Δ Dietary approaches to stop hypertension (DASH)		*p* value
	Decr/maint	T3		Decr/maint	T3		Decr/maint	T3	
	*n* = 1124	*n* = 1423		*n* = 3408	*n* = 534		*n* = 2793	*n* = 852	
Baseline 17-item erMedDiet score, points	10.5 ± 2.5	6.5 ± 1.9	< 0.01	8.7 ± 2.7	7.7 ± 2.6	< 0.01	9 ± 2.7	7.4 ± 2.4	< 0.01
Baseline Trichopoulou MedDiet, points	4.6 ± 1.6	4.2 ± 1.6	< 0.01	5 ± 1.5	2.7 ± 1.3	< 0.01	4.7 ± 1.6	3.8 ± 1.6	< 0.01
Baseline DASH, points	25.3 ± 5.2	22.6 ± 4.9	< 0.01	24.7 ± 5.1	21.9 ± 5	< 0.01	26.2 ± 4.8	19.3 ± 3.8	< 0.01
Age, years	65.3 ± 5	64.6 ± 4.8	< 0.01	65.2 ± 4.9	64.8 ± 4.9	0.09	65.3 ± 4.9	64.7 ± 4.8	< 0.01
Women, % (*n*)	51.4 (578)	43.6 ( 621)	< 0.01	48.2 (1643)	47.6 (254)	0.91	51.2 (1431)	37.8 (322)	< 0.01
BMI, kg/m^2^	32.5 ± 3.5	32.6 ± 3.5	0.19	32.5 ± 3.4	32.6 ± 3.5	0.45	32.5 ± 3.5	32.7 ± 3.5	0.35
PA, METS/min/week	2622.5 ± 2468.1	2375.8 ± 2321.4	0.01	2534.7 ± 2296.4	2745 ± 2615.5	0.04	2597.5 ± 2322.2	2413.6 ± 2202.9	0.16
Energy intake, kcal/d	2347.9 ± 549.5	2423 ± 544	< 0.01	2409.6 ± 542.8	2311.4 ± 551.8	< 0.01	2355.9 ± 533.7	2476.8 ± 559.8	< 0.01
Protein intake, % energy	17 ± 2.8	16.3 ± 2.7	< 0.01	16.6 ± 2.7	17.1 ± 2.9	< 0.01	16.9 ± 2.8	16.4 ± 2.8	< 0.01
Smoking status, % (*n*)			0.39			0.89			0.02
Never smoked	45.3 (509)	43 (612)		44.9 (1530)	42.7 (228)		45.9 (1283)	40.6 (346)	
Former smoker	40.9 (459)	44.6 (634)		42.9 (1463)	43.6 (233)		41.2 (1150)	47.3 (403)	
Current smoker	13.9 (156)	12.4 (177)		12.2 (415)	13.7 (73)		12.9 (360)	12.1 (103)	
Education level, % (*n*)			0.20			0.27			0.02
Primary education	51.4 (578)	48.6 (692)		49.4 (1684)	50.4 (269)		51.1 (1427)	49.4 (421)	
Secondary education	26 (292)	30.9 (439)		28.3 (965)	31.7 (169)		27 (754)	30.6 (261)	
Academic or graduate	22.6 (254)	20.5 (292)		22.3 (759)	18 (96)		21.9 (612)	20 (170)	
eGFR, mL/min/1.73m^2^	83.4 ± 14.4	84.7 ± 13.5	0.12	84.1 ± 13.9	83.6 ± 14.3	0.72	84.4 ± 13.8	84.1 ± 14.2	0.46
CKD, % (*n*)	7.4 (83)	6.1 (87)	0.42	6.6 (224)	7.5 (40)	0.76	6.3 (176)	7 (60)	0.43
Type 2 diabetes, % (*n*)	31.2 (351)	27.1 (385)	0.01	30.7 (1047)	27.5 (147)	0.38	30.8 (859)	28.8 (245)	0.21
Hypertension, % (*n*)	83.9 (943)	85.2 (1212)	0.58	83.8 (2856)	86 (459)	0.02	83.2 (2324)	86 (733)	0.17
Hypercholesterolemia, % (*n*)	68.8 (773)	70.6 (1004)	0.50	69.6 (2372)	70.6 (377)	0.83	70.1 (1959)	69.1 (589)	0.55

Values are presented as percentages (*n*) for categorical variables and means ± standard deviations for continuous variables. *p* value for the comparison among all categories was calculated by chi-square or one-way analysis of variance test for categorical and continuous variables, respectively

*Decr/Maint*, Decrease/Maintenance; *T*, tertile; *MedDiet*, Mediterranean Diet; *BMI*, Body mass index; *PA*, Physical activity; *eGFR*, estimated Glomerular filtration rate; *CKD*, Cronic kidney disease (eGFR < 60 mL/min/1.73m^2^)

The mean eGFR of the study population at baseline was 84.2 mL/min/1.73 m^2^. Over the first year of follow-up, the mean eGFR change was -0.94 mL/min/1.73 m^2^. Multivariable linear regression analyses for the associations between categories of changes in the adherence to the different dietary patterns and changes in eGFR at one year are depicted in Table 2. In the fully adjusted model, one year 17-item erMedDiet score changes were directly associated with eGFR changes (*β*: 0.78; 95% CI: 0.12–1.44 for T1 *vs*. decrease/maintenance; *β*: 1.06; 95% CI: 0.31–1.82 for T2 *vs*. decrease/maintenance; and *β*: 1.87; 95% CI: 1.00–2.73 for T3 *vs*. decrease/maintenance). Similar results were observed when we modeled this dietary pattern as continuous variable (*β*: 0.23; 95% CI: 0.13–0.32 for each 1-point increment). Changes in the Trichopoulou-MedDiet and DASH scores were not associated with eGFR changes neither when analyzed as categories nor in continuous manner, in any of the adjusted models.

**Table 2:** Multivariable adjusted β-coefficients and 95% CI for changes in eGFR (ml/min/1.73m^2^) across categories of changes to the Mediterranean Diet (17-item erMedDiet score and Trichopoulou) and DASH Diet adherence after 1 year of follow-up in the PREDIMED-PLUS cohort

	Δ Mediterranean diet (17-item erMedDiet score)				
	Decr/Maint	T1	T2	T3	*p* for trend
	(*n* = 1124)	(*n* = 1917)	(*n* = 1211)	(*n* = 1423)	
Δ 17-item erMedDiet score	− 1.2 ± 1.4	2.1 ± 0.8	4.5 ± 0.5	7.5 ± 1.6	
Δ eGFR, ml/min/1.73m^2^^a^	− 1.92 (− 2.49 to − 1.34)	− 1.10 (− 1.5 to − 0.71)	− 0.83 (− 1.29 to − 0.37)	− 0.03 (− 0.55–0.48)	
β (95% CI)					
Model 1	0 (Ref.)	0.41 (− 0.23–1.05)	0.54 (− 0.14–1.22)	1.02 (0.35–1.69)^*^	0.003
Model 2	0 (Ref.)	0.45 (− 0.19–1.08)	0.5 (− 0.20–1.19)	0.96 (0.25–1.68)^*^	0.010
Model 3	0 (Ref.)	0.78 (0.12–1.44)^*^	1.06 (0.31 to 1.82)^*^	1.87 (1.00–2.73)^*^	< 0.001

	Δ Mediterranean diet (Trichopoulou)				
	(*n* = 3408)	(*n* = 1055)	(*n* = 678)	(*n* = 534)	
Δ Trichopoulou-MedDiet	− 1.2 ± 1.2	1 ± 0	2 ± 0	3.5 ± 0.7	
Δ eGFR, ml/min/1.73m^2^^a^	− 1.11 (− 1.41 to − 0.81)	− 0.56 (− 1.07 to − 0.05)	− 0.75 (− 1.42 to − 0.08)	− 0.81 (− 1.57 to − 0.05)	
β (95% CI)					
Model 1	0 (Ref.)	0.41 (− 0.17–1.00)	0.23 (− 0.49–0.95)	0.23 (− 0.52–0.98)	0.258
Model 2	0 (Ref.)	0.48 (− 0.1–1.07)	0.25 (− 0.47–0.97)	0.19 (− 0.57–0.95)	0.253
Model 3	0 (Ref.)	0.56 (− 0.05–1.16)	0.35 (− 0.41–1.11)	0.33 (− 0.52–1.18)	0.169

	Δ Dietary approaches to stop hypertension (DASH)				
	(*n* = 2793)	(*n* = 1131)	(*n* = 899)	(*n* = 852)	
Δ DASH	− 3.7 ± 3.3	2.0 ± 0.8	4.9 ± 0.8	9.6 ± 2.7	
Δ eGFR, ml/min/1.73m^2^^a^	− 0.98 (− 1.33 to − 0.62)	− 0.74 (− 1.2 to − 0.28)	− 1.03 (− 1.62 to − 0.45)	− 0.98 (− 1.63 to − 0.33)	
β (95% CI)					
Model 1	0 (Ref.)	0.24 (− 0.32–0.80)	0.07 (− 0.58–0.73)	0.19 (− 0.47–0.86)	0.536
Model 2	0 (Ref.)	0.25 (− 0.31–0.81)	− 0.00 (− 0.67–0.66)	0.06 (− 0.62–0.75)	0.819
Model 3	0 (Ref.)	0.22 (− 0.37− 0.81)	− 0.06 (− 0.78–0.66)	− 0.04 (− 0.84–0.77)	0.957

*Decr/Maint*, Decrease/Maintenance; *T*, tertile; *eGFR*, Estimated glomerular filtration rate; *MedDiet*, Mediterranean Diet; *DASH*, Dietary approaches to stop hypertension

Linear regression models were used to assess changes in eGFR by categories of changes in dietary patterns score. Model 1 was adjusted for baseline eGFR, sex and age. Model 2 was additionally adjusted for BMI, smoking habits (never, current or former smoker), educational level (primary, secondary education, graduate), leisure time physical activity (METS/min/week), diabetes prevalence (yes/no), hypertension prevalence (yes/no), hypercholesterolemia prevalence (yes/no), center (categorized into quartiles by number of participants), intervention group and energy intake (kcal/day). Model 3 was additionally adjusted for each baseline dietary pattern score and 1-year changes in body weight

^a^Multivariable adjusted mean changes in eGFR (ml/min/1.73m^2^) after 1 year of follow-up

*p value < 0.05

No statistically significant interactions were observed between sex, age, diabetes status or intervention group and the changes in the dietary patterns scores mentioned above in relation to changes in eGFR (data not shown).

Table 3 shows the association between changes in dietary patterns and ORs of ≥ 10% eGFR decline after 1 year of follow-up. Changes in the 17-item erMedDiet score showed a significant graded association with eGFR decline in the fully adjusted model (OR: 0.75; 95% CI: 0.61–0.92 for T1 of increase vs. decrease/maintenance; OR: 0.68; 95% CI: 0.53–0.87 for T2 of increase *vs*. decrease/maintenance; OR: 0.62; 95% CI: 0.47–0.82, for T3 of increase *vs*. decrease/maintenance). No significantly associations were observed between changes in the Trichopoulou-MedDiet neither in the DASH score and ≥ 10% eGFR decline after 1 year. When these dietary patterns were modeled as continuous variables, similar associations were obtained, being only the 17-item erMedDiet score associated with lower odds of ≥ 10% eGFR decline (OR: 0.95; 95%: CI 0.92–0.98 for 1-point increment). Similar results were observed after excluding 378 participants with CKD at baseline from the main analysis, which are depicted in Supplementary Table 4.

**Table 3:** Multivariable adjusted odd ratios and 95% CI for eGFR decline (> 10%) across categories of changes to the Mediterranean Diet (17-item erMedDiet score and Trichopoulou) and DASH Diet after 1 year of follow-up

	Δ Mediterranean diet (17-item erMedDiet score)				
	Decr/Maint	T1	T2	T3	*p* for trend
	(*n* = 1124)	(*n* = 1917)	(*n* = 1211)	(*n* = 1423)	
Δ 17-item erMedDiet score	− 1.2 ± 1.4	2.1 ± 0.8	4.5 ± 0.5	7.5 ± 1.6	
eGFR decline, % (*n*)	18.33 (206)	15.86 (304)	15.03 (182)	15.11 (215)	
Model 1	1 (Ref.)	0.84 (0.69–1.02)	0.80 (0.64–0.99)^*^	0.80 (0.65 to 0.99)^*^	0.052
Model 2	1 (Ref.)	0.81 (0.66–0.99)^*^	0.77 (0.61–0.97)^*^	0.76 (0.60 to 0.96)^*^	0.029
Model 3	1 (Ref.)	0.75 (0.61–0.92)^*^	0.68 (0.53–0.87)^*^	0.62 (0.47 to 0.82)^*^	0.001

	Δ Mediterranean diet (Trichopoulou)				
	(*n* = 3408)	(*n* = 1055)	(*n* = 678)	(*n* = 534)	
Δ Trichopoulou-MedDiet	− 1.2 ± 1.2	1 ± 0	2 ± 0	3.5 ± 0.7	
eGFR decline, % (*n*)	16.08 (548)	16.30 (172)	14.90 (101)	16.10 (86)	
Model 1	1 (Ref.)	1.02 (0.85–1.24)	0.92 (0.73–1.16)	1.01 (0.79–1.29)	0.809
Model 2	1 (Ref.)	1.00 (0.83–1.21)	0.91 (0.72–1.15)	1.00 (0.77–1.29)	0.686
Model 3	1 (Ref.)	1.02 (0.84–1.25)	0.94 (0.73–1.20)	1.05 (0.79–1.39)	0.998

	Δ Dietary approaches to stop hypertension (DASH)				
	(*n* = 2793)	(*n* = 1131)	(*n* = 899)	(*n* = 852)	
Δ DASH	− 3.7 ± 3.3	2.0 ± 0.8	4.9 ± 0.8	9.6 ± 2.7	
eGFR decline, % (*n*)	16.36 (457)	14.41 (163)	17.02 (153)	15.73 (134)	
Model 1	1 (Ref.)	0.87 (0.72–1.05)	1.06 (0.87–1.30)	0.97 (0.78–1.20)	0.872
Model 2	1 (Ref.)	0.85 (0.70–1.04)	1.04 (0.84–1.28)	0.95 (0.76–1.18)	0.685
Model 3	1 (Ref.)	0.85 (0.70–1.05)	1.05 (0.83–1.32)	0.97 (0.74–1.26)	0.862

Logistic regression models were used to assess eGFR decline (> 10%) by categories of dietary patterns score changes. Model 1 was adjusted for sex and age. Model 2 was additionally adjusted for BMI, smoking habits (never, current or former smoker), educational level (primary, secondary education, graduate), leisure time physical activity (METS/min/week), diabetes prevalence (yes/no), hypertension prevalence (yes/no), hypercholesterolemia prevalence (yes/no), center (categorized into quartiles by number of participants), intervention group and energy intake (kcal/day). Model 3 was additionally adjusted for each baseline dietary pattern score and 1-year changes in body weight

*Decr/Maint*, Decrease/Maintenance; *T*, tertile; *eGFR*, Estimated glomerular filtration rate; *MedDiet*, Mediterranean Diet; *DASH*, Dietary approaches to stop hypertension

*p value < 0.05

**Fig. 1:**
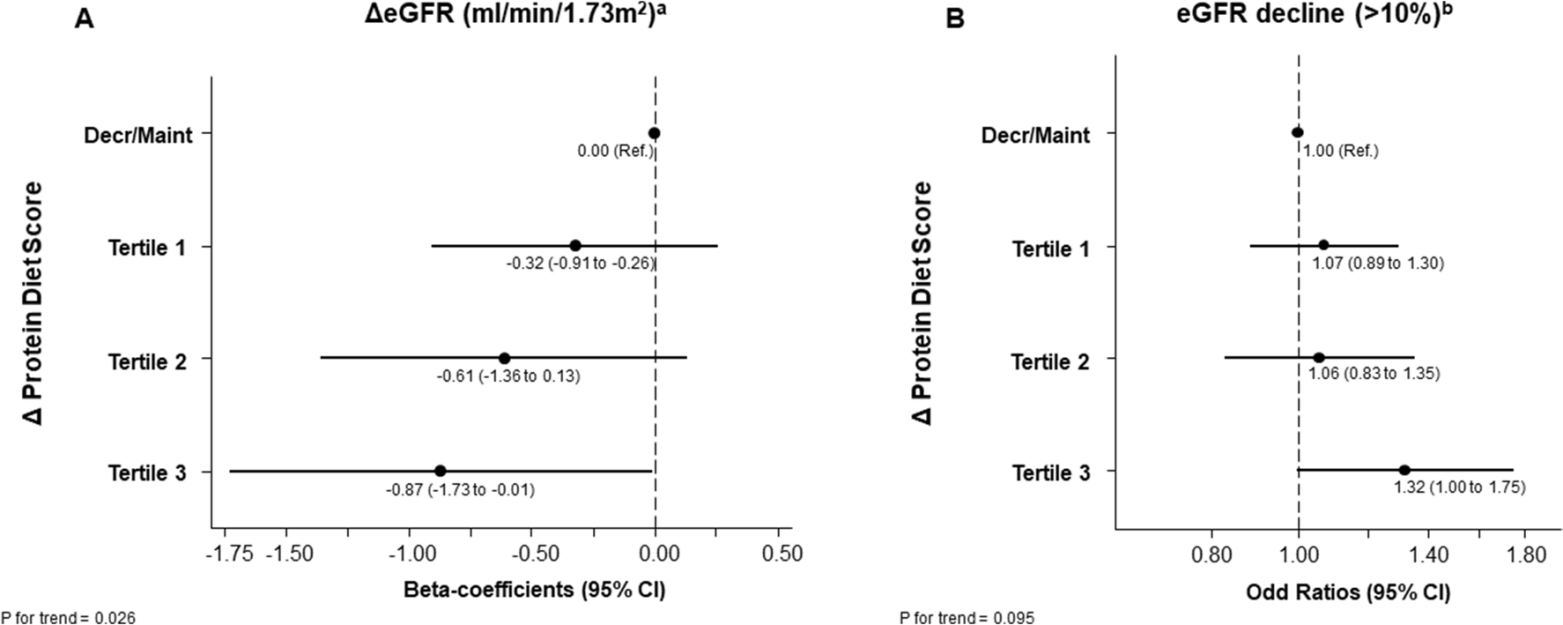
Associations between changes in Protein Diet score and kidney function. **A** Multivariable adjusted β-coefficients and 95%CI for changes in eGFR (ml/min/1.73m^2^) across categories of changes to the protein diet score adherence after 1 year of follow-up. **B** Multivariable adjusted odd ratios and 95%CI for eGFR decline (> 10%) across categories of changes to the protein diet score adherence after 1 year of follow-up. *Decr/Maint*, Decrease/Maintenance; *eGFR*, Estimated glomerular filtration rate; *Decr/Maint*, Decrease/Maintenance. ^a^Mean changes in eGFR (ml/min/1.73m^2^): Decr/Maint (− 0.71; − 1.02 to − 0.41), tertile 1 of changes (− 1.05; − 1.52 to − 0.57), tertile 2 of changes (− 1.31; − 1.96 to − 0.65), tertile 3 of changes (− 1.61; − 2.38 to − 0.84). ^b^Percentage of participants with eGFR decline(> 10%): Decr/Maint (*n* = 507; % = 15.54), tertile 1 of changes (*n* = 196; % = 16.27), tertile 2 of changes (*n* = 106; % = 15.57), tertile 3 of changes (*n* = 98; % = 18.60). All models were adjusted for baseline eGFR (except for eGFR decline > 10%), sex, age, BMI, smoking habits (never, current or former smoker), educational level (primary, secondary education, graduate), leisure time physical activity (METS/min/week), diabetes prevalence (yes/no), hypertension prevalence (yes/no), hypercholesterolemia prevalence (yes/no), center (categorized into quartiles by number of participants), intervention group and energy intake (kcal/day) and 1-year changes in body weight

Figure 1 displays the association between changes in Protein Diet score and eGFR changes and ≥ 10% eGFR decline after one year of follow-up. We observed a significant association between this score and both renal outcomes. Compared to participants without changes or with a decrease in the Protein Diet score, those in the highest tertile of increase had greater downward changes in eGFR (*β*: − 0.87; 95% CI: − 1.73 to − 0.01) and a 32% higher odds of eGFR decline (OR: 1.32; 95% CI: 1.00–1.75) in the fully adjusted model. When each component of this score was examined separately, only the change in total protein intake (E%) score presented a significant inverse association with eGFR changes (*β*: − 1.04; 95% CI: − 1.88 to − 0.21; for T3 *vs*. decrease/maintenance) in the multivariate adjusted model.

Table 4 shows the additional analysis after the alternatively removal of each individual component of the 17-item erMedDiet score modeled as continuous in the regression models. The results remained consistent with those from the main analyses for both eGFR changes and ≥ 10% eGFR decline. We observed that the greatest contributors to the association between changes in 17-item erMedDiet score and eGFR changes after 1 year of follow-up were the consumption of ≥ 2 units/day of vegetables, ≥ 3 servings/week of legumes (13% reduction in the association after removing these items from the total score); the use of sofrito ≥ 2 times/week (17% reduction) and moderate consumption of wine (22% reduction). Similar results were obtained when we performed the same removing items procedures for ≥ 10% eGFR decline as an outcome variable.

**Table 4:** Association between 1-point increment in the 17-item erMedDiet score and changes in eGFR (ml/min/1.73m^2^) and eGFR decline (> 10%) after alternate subtraction of each of its dietary components

	β-coefficient (95%CI Δ eGFR	Reduction/increase (%)	OR (95%CI) eGFR decline (> 10%)	Reduction/increase (%)
17-item erMedDiet score overall	0.23 (0.13–0.32)	–	0.95 (0.92–0.98)	–
Minus item 1	0.21 (0.12–0.30)	− 8.70	0.96 (0.93–0.98)	− 20
Minus item 2	0.20 (0.11–0.30)	− 13.04	0.96 (0.93–0.99)	− 20
Minus item 3	0.22 (0.12–0.31)	− 4.35	0.96 (0.93–0.99)	− 20
Minus item 4	0.24 (0.14–0.33)	4.35	0.96 (0.92–0.99)	− 20
Minus item 5	0.22 (0.13–0.31)	− 4.35	0.95 (0.93–0.98)	0
Minus item 6	0.22 (0.12–0.31)	− 4.35	0.96 (0.93–0.98)	− 20
Minus item 7	0.20 (0.10–0.29)	− 13.04	0.96 (0.93–0.99)	− 20
Minus item 8	0.22 (0.13–0.31)	− 4.35	0.95 (0.92–0.98)	0
Minus item 9	0.24 (0.15–0.34)	4.35	0.95 (0.92–0.98)	0
Minus item 10	0.24 (0.15–0.34)	4.35	0.95 (0.92–0.98)	0
Minus item 11	0.21 (0.12–0.31)	− 8.70	0.95 (0.92–0.98)	0
Minus item 12	0.18 (0.09–0.28)	− 17.39	0.96 (0.93–0.99)	− 20
Minus item 13	0.24 (0.15–0.34)	4.35	0.95 (0.92–0.98)	0
Minus item 14	0.23 (0.13–0.33)	0	0.95 (0.92–0.98)	0
Minus item 15	0.22 (0.12–0.32)	− 4.35	0.95 (0.92–0.98)	0
Minus item 16	0.28 (0.18–0.38)	13.04	0.93 (0.90–0.96)	40
Minus item 17	0.20 (0.11–0.29)	− 21.73	0.96 (0.93–0.99)	− 20

Models were adjusted for baseline eGFR (only for β-coefficient), sex, age, BMI, smoking habits (never, current or former smoker), educational level (primary, secondary education, graduate), leisure time physical activity (METS/min/week), diabetes prevalence (yes/no), hypertension prevalence (yes/no), hypercholesterolemia prevalence (yes/no), center (categorized into quartiles by number of participants), intervention group and energy intake (kcal/day), 1-year changes in body weight, baseline dietary pattern score and changes in corresponding subtracted components

*eGFR*, Estimated glomerular filtration rate; *MedDiet*, Mediterranean diet; *Item 1*, use only extra virgin olive oil for cooking; *Item 2*, consume ≥ 2 of vegetables units/day; *Item 3*, consume ≥ 3 fruits units/day; *Item 4*, consume ≤ 1 serving/week of red meat and processed meats; *Item 5*, consume < 1 serving/week of butter, margarine or cream; *Item 6*, drink < 1 sugar-sweetened beverage or fruit juice/week; *Item 7*, consume ≥ 3 servings/week of legumes; *Item 8*, consume ≥ 3 servings/week of fish or shellfish; *Item 9*, consume < 3 times/week commercial sweets or pastries; *Item 10*, consume ≥ 3 servings/week of nuts; *Item 11*, consume preferentially lean meats; *Item 12*, consume ≥ 2 times/week dishes seasoned with sofrito (tomato, onion, leek or garlic sauce simmered in olive oil); *Item 13*, add preferentially noncaloric artificial sweeteners to beverages; *Item 14*, consume ≥ 1 servings/day of white bread; *Item 15*, consume ≥ 5 times/week whole grain cereals and pasta; *Item 16*, consume < 3 times/week refined pasta or white rice; *Item 17*, moderate consume of wine /day (2–3 glasses in men/1–2 in women)

## Discussion

To the best of our knowledge, this is the first prospective study examining the association between different a priori defined dietary patterns adherence and kidney function in elderly individuals with overweight/obesity and MetS. We found that a higher upward change in the adherence to a 17-item erMedDiet score was associated with improvements in eGFR changes and with 38% lower odds of eGFR decline after controlling for potential confounders. However, changes in the adherence to the Trichopoulou-MedDiet and DASH Score were not associated with changes in eGFR, neither with a ≥ 10% eGFR decline. Regarding the Protein Diet Score, higher changes toward a greater adherence were associated with a worsening of eGFR.

Previous studies have reported that MedDiet adherence is inversely associated with eGFR decline [31], incidence of CKD [32, 33], and risk of end-stage of kidney disease (ESKD) [34] in populations of youth to middle-age with apparently preserved eGFR. Our results regarding the pre-specified 17-item Med Diet score are in line with two prior randomized clinical trials, the PREDIMED in Spain [35] and the DIRECT [36] in Israel, in which MedDiet improved levels of eGFR in participants with overweight/obesity, whereas the data-driven by the Trichopoulou score not. In the Lifelines cohort study conducted on apparently healthy middle-aged adults of Netherlands, an inverse association between the MedDiet adherence according to Trichopoulou and eGFR decline was reported in men, but not in women [31]. Besides, in the Uppsala Longitudinal Study of Adult Men cohort (ULSAM), conducted in middle-age men, it was observed that a greater adherence to the MedDiet according Trichopoulou was significantly associated with lower odds of having CKD when it was estimated by cystatine C, but not by creatinine estimation [33]. In our study, the analysis was performed in the whole study population because we did not observe any interaction with sex. However, considering that previous evidence has reported significant associations in men but not in women, future studies stratifying by sex are needed to shed new light on whether the MedDiet could have a different role in kidney function in men than women. In the Northern Manhattan Study (NOMAS), a prospective multiethnic cohort conducted in 3298 middle-age participants with no previous history of stroke, no significant association were observed across their four categories of Trichopoulou-MedDiet adherence and eGFR decline, neither change in eGFR [15]. The apparent disagreement between 17-MedDiet and Trichopoulou-MedDiet Scores in our study reveals the disparities between both tools concerning the items included. The inclusion of more food groups into the 17-MedDiet Score screener tool maybe would imply a more representative assessment of diet to observe changes in eGFR. Additionally, scoring criteria are quite different; while the 17-item erMedDiet uses absolute values derived from a predefined questionnaire, the Trichopoulou-MedDiet score is assigned according to gender-specific medians of food group consumption of the study population [26]. The later may hinder comparability with other publications where particular cultural and dietary habits are present. Likewise, these issues may explain the controversial results observed between both MedDiet scoring methods in our sample. Of note, this is the first study so far analyzing the association between the adherence to the MedDiet, using the 17-MedDiet score screener tool, and renal function. Further studies, considering both scores (Trichopoulou-MedDiet and 17-item erMedDiet) are needed to clarify and strengthen our results.

Although the DASH diet has been formerly associated with better kidney function outcomes such as rapid eGFR decline [13] or CKD incidence [37], a recent meta-analysis did not confirm such associations [11], which is partly agreed with our findings. In fact, in the Healthy Aging in Neighborhoods of Diversity across the Life Span (HANDLS) study [16], the authors not only reported a lack of association between this dietary pattern and eGFR decline or incident CKD but also even greater risk of rapid kidney function decline among the group of middle-age individuals with hypertension after 5 years of follow-up. Whether a plant-based diet like the DASH, which is apparently similar to the MedDiet, is not associated with a better kidney function, despite being protective against hypertension [38], which in turn is a well-known risk factor for kidney disease, needs to be further investigated.

The underlying biological mechanisms whereby changes toward a greater adherence to a plant-based diet as the MedDiet, but not the DASH diet, could preserve or improve kidney function are not entirely clear. When we analyzed individual components of the 17-item erMedDiet Score, vegetables, legumes, wine and the traditional Mediterranean tomato and olive oil sauce (*sofrito*) were associated with better renal function. These foods represent the main differences between the two dietary aforementioned patterns and are rich in some beneficial nutrients such as fiber, antioxidants and anti-inflammatory compounds that may play a protective role by reducing the levels of inflammatory biomarkers, improving endothelial function, plasma lipid profiles and insulin resistance, lowering high blood pressure, and preserving a low glycemic index and load [6, 39–45].

Both Mediterranean diet and DASH, which has been usually associated with better markers of kidney function, are rich in plant-protein. Despite some evidence has raised concerns about the detrimental effects of high-protein intake on kidney damage [46], it seems that besides the quantity, the source of protein intake might be considered when analyzing these associations. Likewise, its long-term effects in vulnerable elderly individuals are still unknown. Our results regarding the Protein Diet Score are not in line with those of a previous cross-sectional study based on three cohort [18], NQplus, Lifelines, and the Young Finns Study, where a positive association between the protein score and eGFR was repy, by assuming that participants with renal dysfunction have already changed their protein intake [18]. Besides, our results from each component of the Protein Diet Score suggest that increased protein total intake could be the major drive for the deleterious renal association observed. Further research with long duration is warranted using this score to clarify its potential implication in kidney function and damage.

Limitations of the current study must be considered when interpreting the results. Firstly, eGFR measurement was estimated using SCr, as in most of epidemiologic studies, but other more optimal biomarkers exist. However, the procedures of those biomarkers are expensive, time consuming and difficult to be used in large population studies. Secondly, although the FFQ is an appropriate tool to assess usual dietary intake when it is carefully administered by trained staff, recall bias could not be ruled out. Thirdly, this study was conducted in elderly Mediterranean individuals with overweight/obesity and MetS; consequently, our findings cannot be extrapolated to other study populations. Fourthly, PREDIMED-Plus study is a randomized controlled trial; therefore, the lifestyle advice in turn could affect our findings. Nevertheless, we adjusted our analyses by the intervention group. Finally, SCr has a well-known biological variability and as we only measured it at baseline and 1 year (at short term), some degree of misclassification could be present. The present study also has notable strengths, which deserve to be mentioned, such as its prospective design, the relatively large sample size and the inclusion of different dietary patterns in main analyses. Moreover, we adjusted our models for a substantial number of covariates which could affect renal function, to try to control for potential bias. Even so, as in any observational study, we cannot rule out the possibility of residual or unmeasured confounding.

## Conclusion

In summary, changes towards a greater adherence in the 17-item erMedDiet score after 1 year of follow-up were associated with better eGFR and lower odds of ≥ 10% eGFR decline in an elderly population with MetS. Not significant results were observed with regards to the Trichopoulou-MedDiet and DASH Score. These discrepancies could be partially explained by their differences in the calculation of the score, which in contrast to the 17-item erMedDiet depends on cut-off points based on the study population distribution of each item. Future studies in the renal function field should consider including the 17-item erMedDiet score in their analyses to clarify and strengthen our findings. Besides, the Protein Diet Score was associated with changes towards a worse eGFR and higher odds of ≥ 10% eGFR decline. Our results provide further insights to the evidence concerning a priori dietary patterns associated with kidney function in populations at high cardiovascular risk and reinforce the role of a plant-protein-based healthy diet in preserving renal function, particularly among this vulnerable population group. Therefore, improving dietary habits following a MedDiet could lead to a better kidney function, and even it could be considered an appropriate and safe preventive strategy against the onset or progression of CKD. However, these findings should be confirmed by future long-term studies and randomized controlled trials before including this kind of diet in the prevention and management guidelines of CKD.

## Supplementary Information

Below is the link to the electronic supplementary material.

Supplementary file1 (DOCX 48 kb)

## Abbreviations

BMI: Body mass index
CKD: Chronic kidney disease
CI: Confidence interval
DASH: Dietary approach to stop hypertension
E: Energy
FFQ: Food frequency questionnaire
GFR: Glomerular filtration rate
MedDiet: Mediterranean diet
MetS: Metabolic syndrome
METS: Metabolic equivalent task
PREDIMED: Prevención con dieta Mediterránea
SCr: Serum creatinine
CKD-EPI: Chronic kidney disease epidemiology collaboration equation for caucasian individuals
OR: Odds ratios

## Declarations

### Availability of the data and materials

There are restrictions on the availability of data for the PREDIMED-Plus trial, due to the signed consent agreements around data sharing, which only allow access to external researchers for studies following the project purposes. Requestors wishing to access the PREDIMED-Plus trial data used in this study can make a request to the PREDIMED-Plus trial Steering Committee chair: predimed_plus_scommitte@googlegroups.com. The request will then be passed to members of the PREDIMED-Plus Steering Committee for deliberation.
