# Supplementary material for: Prospective associations between a priori dietary patterns adherence and kidney function in an elderly Mediterranean population at high cardiovascular risk
Source: Eur J Nutr. 2022 Apr 2;61(6):3095–108. doi: 10.1007/s00394-022-02838-7 (PMC9363380; doi:10.1007/s00394-022-02838-7)
Supplement: Supplementary file 1 — Supplementary file1 (DOCX 48 kb) [file 394_2022_2838_MOESM1_ESM.docx]

**Prospective associations between a priori dietary patterns adherence and kidney function in an elderly Mediterranean population at high cardiovascular risk**

**EUROPEAN JOURNAL OF NUTRITION**

Cristina Valle-Hita^1,2,3⁑^; Andrés Díaz-López^2,4,5⁑^; Nerea Becerra-Tomás^1,2,4,6*^; Miguel A. Martínez-González^4,7,8^; Verónica Ruiz García^1,9^; Dolores Corella^4,10^; Albert Goday^4,11^; J. Alfredo Martínez^4,12,13^; Ángel M. Alonso-Gómez^4,15^; Julia Wärnberg^4,16^; Jesús Vioque^17,18^; Dora Romaguera^4,19^; José López-Miranda^,4,20^; Ramon Estruch^4,21^; Francisco J. Tinahones^4,22^; José Lapetra^4,23^; Luís Serra-Majem^4,24^; Naomi Cano-Ibañez^18,25^; Josep A. Tur^4,19,22^; María Rubín-García^27^; Xavier Pintó^4,28,29^; Miguel Delgado-Rodríguez^18,30^; Pilar Matía-Martín^31^; Josep Vidal^32,33^; Sebastian Mas Fontao^34^; Lidia Daimiel^n^; Emilio Ros^4,35^; Estefania Toledo^4,7^; José V Sorlí^4,10^; C Roca^4,11^; Iztiar Abete^4,12^; Anai Moreno-Rodriguez^4,15^; Edelys Crespo-Oliva^4,16^; Inmaculada Candela-García^36^; Marga Morey^4,19^; Antonio Garcia-Rios^4,20^; Rosa Casas^4,21^; Jose Carlos Fernandez-Garcia^22^; José Manuel Santos-Lozano^4,23^; Javier Diez-Espino^4,7,37^; Carolina Ortega-Azorín^4,10^; M Comas^4,11^; M. Angeles Zulet^4,12^; Carolina Sorto-Sanchez^4,15^; Miguel Ruiz-Canela^4,7^; Montse Fitó^4,11^; Jordi Salas-Salvadó^1,2,3,4^ and Nancy Babio^1,2,3,4^

^1^Universitat Rovira i Virgili, Department of Biochemistry and Biotechonology, Human Nutrition Unit, 43201 Reus, Spain;

^2^Institut d'Investigació Sanitària Pere Virgili (IISPV), 43204 Reus, Spain;

^3^University Hospital of Sant Joan de Reus, Nutrition Unit, 43201 Reus, Spain;

^4^Consorcio CIBER, M.P. Fisiopatología de la Obesidad y la Nutrición (CIBERObn), Institute of Health Carlos III, 28029 Madrid, Spain;

^5^Serra Hunter Fellow, Universitat Rovira i Virgili, Nutrition and Mental Health Research Group (NUTRISAM), 43201 Reus, Spain;

^6^MRC Centre for Environment and Health, Department of Epidemiology & Biostatistics, School of Public Health, Faculty of Medicine, Imperial College London, St Mary's Campus, Norfolk Place, London W2 1PG;

^7^University of Navarra, Department of Preventive Medicine and Public Health, IdiSNA, 31008 Pamplona, Spain;

^8^Department of Nutrition, Harvard T.H. Chan School of Public Health, Boston, MA 02115, USA;

^9^University Hospital of Tarragona Joan XXIII, 43005, Tarragona, Spain;

^10^Department of Preventive Medicine, University of Valencia, 46010 Valencia, Spain;

^11^Cardiovascular Risk and Nutrition research group (CARIN), Hospital del Mar Research Institute (IMIM), 08003 Barcelona, Spain;

^12^University of Navarra, Department of Nutrition, Food Science and Physiology, IdiSNA, 31008 Pamplona, Spain;

^13^Precision Nutrition Program, IMDEA Food, CEI UAM + CSIC, 28049 Madrid, Spain;

^14^Nutritional Control of the Epigenome Group, IMDEA Food, CEI UAM + CSIC, 28049 Madrid, Spain;

^15^Bioaraba Health Research Institute; Osakidetza Basque Health Service, Araba University Hospital; University of the Basque Country UPV/EHU; 01009 Vitoria-Gasteiz, Spain;

^16^Department of Nursing, University of Málaga, Institute of Biomedical Research in Malaga (IBIMA), 29071 Málaga, Spain;

^17^Instituto de Investigación Sanitaria y Biomédica de Alicante, Miguel Hernandez University (ISABIAL-UMH), 46020 Alicante, Spain;

^18^CIBER de Epidemiología y Salud Pública (CIBERESP), Instituto de Salud Carlos III, 28029 Madrid, Spain;

^19^Health Research Institute of the Balearic Islands (IdISBa), 07120 Palma de Mallorca, Spain;

^20^Department of Internal Medicine, Maimonides Biomedical Research Institute of Cordoba (IMIBIC), Reina Sofia University Hospital, University of Cordoba, 14004 Cordoba, Spain;

^21^Department of Internal Medicine, Institutd’Investigacions Biomèdiques August Pi Sunyer (IDIBAPS), Hospital Clinic, University of Barcelona, 08036 Barcelona, Spain;

^22^Virgen de la Victoria Hospital, Department of Endocrinology. Instituto de Investigación Biomédica de Málaga (IBIMA). University of Málaga, 29010 Málaga, Spain;

^23^Department of Family Medicine, Research Unit, Distrito Sanitario Atención Primaria Sevilla, 41013 Sevilla, Spain;

^24^University of Las Palmas de Gran Canaria, Research Institute of Biomedical and Health Sciences (IUIBS), Preventive Medicine Service, Centro Hospitalario Universitario Insular Materno Infantil (CHUIMI), Canarian Health Service, 35016 Las Palmas, Spain;

^25^Department of Preventive Medicine, University of Granada, 18071 Granada, Spain;

^26^Research Group on Community Nutrition & Oxidative Stress, University of Balearic Islands, 07122 Palma de Mallorca, Spain;

^27^Institute of Biomedicine (IBIOMED), University of León, 24071 León, Spain;

^28^Lipids and Vascular Risk Unit, Internal Medicine, Hospital Universitario de Bellvitge-IDIBELL, Hospitalet de Llobregat, 08907 Barcelona Spain;

^29^University of Barcelona. 08007 Barcelona Spain;

^30^Division of Preventive Medicine, Faculty of Medicine, University of Jaén, 23071 Jaén, Spain; ^31^Department of Endocrinology and Nutrition, Instituto de Investigación Sanitaria Hospital Clínico San Carlos (IdISSC), 27040 Madrid, Spain;

^32^Departament of Endocrinology, IDIBAPS, Hospital Clínic, University of Barcelona, 08036 Barcelona, Spain;

^33^CIBER Diabetes y Enfermedades Metabólicas (CIBERDEM), Instituto de Salud Carlos III (ISCIII), 28029 Madrid, Spain;

^34^Department of Endocrinology and Nutrition, University Hospital Fundación Jimenez Díaz, Instituto de Investigaciones Biomédicas IISFJD,28040 Madrid, Spain;

^35^Lipid Clinic, Department of Endocrinology and Nutrition, Institut d’Investigacions Biomèdiques August Pi Sunyer (IDIBAPS), Hospital Clínic, 08036 Barcelona, Spain.

^36^Centro de Salud Santa Pola, 03130 Alicante, Spain

^37^Atención Primaria, Servicio Navarro de Salud, Osasunbidea, Pamplona, Spain.

^⁑^These authors contributed equally to this work.

***Correspondence to:** Dr. Nerea Becerra-Tomás, RD, PhD. Department of Biochemistry and Biotechonology, Human Nutrition Unit, Universitat Rovira i Virgili, Carrer Sant Llorenç, 21 43201 Reus (Spain). E-mail address: nerea.becerra@urv.cat.

**ELECTRONIC SUPPLEMENTARY MATERIAL**

**Supplementary table 1.** Baseline characteristics according to categories of changes in the Mediterranean diet (17-item erMedDiet score and Trichopoulou) adherence after 1 year of follow-up in the PREDIMED-PLUS cohort

**Supplementary table 2.** Baseline characteristics according to categories of changes in the Dietary Approaches to Stop Hypertension (DASH) and the Protein Diet adherence after 1 year of follow-up in the PREDIMED-PLUS cohort

**Supplementary table 3.** Baseline characteristics of excluded vs included individuals for analysis

**Supplementary table 4.** Multivariable adjusted β-coefficients and 95% CI for changes in eGFR (ml/min/1.73m2) and multivariable adjusted odd ratios and 95% CI for eGFR decline (>10%) across categories of changes to the Mediterranean Diet (17-item erMedDiet score and Trichopoulou) and DASH Diet adherence in participants without CKD (eGFR >60 ml/min/1.73m2) at baseline after 1 year of follow-up (n=5297)

| **Supplementary table 1. Baseline characteristics according to categories of changes in the Mediterranean diet (17-item erMedDiet score and Trichopoulou) adherence after 1 year of follow-up in the PREDIMED-PLUS cohort** | | | | | | | | | | | | | | | | | | |  |
| --- | --- | --- | --- | --- | --- | --- | --- | --- | --- | --- | --- | --- | --- | --- | --- | --- | --- | --- | --- |
|  | **Δ Mediterranean Diet (17-item erMedDiet score)** | | | | | | | |  | **Δ Mediterranean Diet (Trichopoulou)** | | | | | | |  | |  |
|  | **Dec/Maint** | **T1** | | **T2** | | **T3** | | |  | **Dec/Maint** | **T1** | | **T2** | | **T3** | |  | |  |
|  | n=1124 | n=1917 | | n=1211 | | n=1423 | | | p-value | n=3408 | n=1055 | | n=678 | | n=534 | | p-value | |  |
| Baseline 17-item erMedDiet score, points | 10.5 ± 2.5 | 9.1 ± 2.4 | | 8 ± 2.2 | | 6.5 ± 1.9 | | | <0.01 | 8.7 ± 2.7 | 8.3 ± 2.6 | | 8.1 ± 2.6 | | 7.7 ± 2.6 | | <0.01 | |  |
| Baseline Trichopoulou-MedDiet, points | 4.6 ± 1.6 | 4.5 ± 1.6 | | 4.3 ± 1.7 | | 4.2 ± 1.6 | | | <0.01 | 5 ± 1.5 | 3.9 ± 1.3 | | 3.4 ± 1.3 | | 2.7 ± 1.3 | | <0.01 | |  |
| Baseline DASH, points | 25.3 ± 5.2 | 24.5 ± 5.2 | | 23.7 ± 5.1 | | 22.6 ± 4.9 | | | <0.01 | 24.7 ± 5.1 | 23.4 ± 5.2 | | 22.7 ± 5.2 | | 21.9 ± 5 | | <0.01 | |  |
| Baseline Protein Diet, points | 10.3 ± 3 | 10 ± 3 | | 10 ± 3 | | 9.7 ± 2.9 | | | <0.01 | 10.3 ± 3 | 9.9 ± 3 | | 9.6 ± 2.9 | | 9.1 ± 2.9 | | <0.01 | |  |
| Age, years | 65.3 ± 5 | 65.3 ± 4.8 | | 64.9 ± 4.9 | | 64.6 ± 4.8 | | | <0.01 | 65.2 ± 4.9 | 64.8 ± 4.8 | | 64.8 ± 4.9 | | 64.8 ± 4.9 | | 0.09 | |  |
| Women, % (n) | 51.4 (578) | 49.5 (949) | | 47.3 (573) | | 43.6 ( 621) | | | <0.01 | 48.2 (1643) | 48.1 (507) | | 46.8 (317) | | 47.6 (254) | | 0.91 | |  |
| BMI, kg/m^2^ | 32.5 ± 3.5 | 32.4 ± 3.4 | | 32.5 ± 3.4 | | 32.6 ± 3.5 | | | 0.19 | 32.5 ± 3.4 | 32.4 ± 3.3 | | 32.7 ± 3.5 | | 32.6 ± 3.5 | | 0.45 | |  |
| PA, METS/min/week | 2622.5 ± 2468.1 | 2618.7 ± 2280.7 | | 2519.6 ± 2316.8 | | 2375.8 ± 2321.4 | | | 0.01 | 2534.7 ± 2296.4 | 2553.8 ± 2456 | | 2361.6 ± 2108.2 | | 2745 ± 2615.5 | | 0.04 | |  |
| Energy intake, kcal/d | 2347.9 ± 549.5 | 2344.7 ± 542.8 | | 2373.8 ± 563.3 | | 2423 ± 544 | | | <0.01 | 2409.6 ± 542.8 | 2311 ± 544.2 | | 2318.9 ± 574.2 | | 2311.4 ± 551.8 | | <0.01 | |  |
| Protein intake, % energy | 17 ± 2.8 | 16.9 ± 2.9 | | 16.8 ± 2.8 | | 16.3 ± 2.7 | | | <0.01 | 16.6 ± 2.7 | 16.9 ± 2.8 | | 17 ± 3 | | 17.1 ± 2.9 | | <0.01 | |  |
| Smoking status, % (n) |  |  | |  | |  | | | 0.39 |  |  | |  | |  | | 0.89 | |  |
| Never smoked | 45.3 (509) | 45.5 (872) | | 43.5 (527) | | 43 (612) | | |  | 44.9 (1530) | 43.6 (460) | | 44.5 (302) | | 42.7 (228) | |  | |  |
| Former smoker | 40.9 (459) | 42.1 (807) | | 44.4 (538) | | 44.6 (634) | | |  | 42.9 (1463) | 43.1 (455) | | 42.3 (287) | | 43.6 (233) | |  | |  |
| Current smoker | 13.9 (156) | 12.4 (238) | | 12.1 (146) | | 12.4 (177) | | |  | 12.2 (415) | 13.3 (140) | | 13.1 (89) | | 13.7 (73) | |  | |  |
| Education level, % (n) |  |  | |  | |  | | | 0.20 |  |  | |  | |  | | 0.27 | |  |
| Primary education | 51.4 (578) | 49.5 (948) | | 49.5 (599) | | 48.6 (692) | | |  | 49.4 (1684) | 50.8 (536) | | 48.4 (328) | | 50.4 (269) | |  | |  |
| Secondary education | 26 (292) | 28.6 (548) | | 29.9 (362) | | 30.9 (439) | | |  | 28.3 (965) | 29.1 (307) | | 29.5 (200) | | 31.7 (169) | |  | |  |
| Academic or graduate | 22.6 (254) | 22 (421) | | 20.6 (250) | | 20.5 (292) | | |  | 22.3 (759) | 20.1 (212) | | 22.1 (150) | | 18 (96) | |  | |  |
| eGFR, mL/min/1.73m^2^ | 83.4 ± 14.4 | 84.1 ± 13.9 | | 84.3 ± 14.2 | | 84.7 ± 13.5 | | | 0.12 | 84.1 ± 13.9 | 84.4 ± 13.9 | | 84.4 ± 14 | | 83.6 ± 14.3 | | 0.72 | |  |
| CKD, % (n) | 7.4 (83) | 6.3 (120) | | 7.3 (88) | | 6.1 (87) | | | 0.42 | 6.6 (224) | 6.9 (73) | | 6.1 (41) | | 7.5 (40) | | 0.76 | |  |
| Type 2 diabetes, % (n) | 31.2 (351) | 32.1 (616) | | 31.1 (376) | | 27.1 (385) | | | 0.01 | 30.7 (1047) | 31.6 (333) | | 29.7 (201) | | 27.5 (147) | | 0.38 | |  |
| Hypertension, % (n) | 83.9 (943) | 84.4 (1618) | | 83.2 (1008) | | 85.2  (1212) | | | 0.58 | 83.8 (2856) | 82.7 (872) | | 87.6 (594) | | 86 (459) | | 0.02 | |  |
| Hypercholesterolemia, % (n) | 68.8 (773) | 70.3 (1347) | | 68.3 (827) | | 70.6 (1004) | | | 0.50 | 69.6 (2372) | 70.1 (739) | | 68.3 (463) | | 70.6 (377) | | 0.83 | |  |
| Abbreviations: Decr/Maint, Decrease/Maintenance; MedDiet, Mediterranean Diet; BMI, Body Mass Index; PA, Physical activity; eGFR, estimated Glomerular Filtration Rate; CKD, Chronic Kidney Disease (eGFR<60 mL/min/1.73m^2^).  Values are presented as percentages (n) for categorical variables and means ± standard deviations (SD) for continuous variables. P-value was calculated by chi-square or one-way analysis of variance test for categorical and continuous variables, respectively. | | | | | | | | | | | | | | | | | | |  |
| **Supplementary table 2. Baseline characteristics according to categories of changes in the Dietary Approaches to Stop Hypertension (DASH) and the Protein Diet adherence after 1 year of follow-up in the PREDIMED-PLUS cohort** | | | | | | | | | | | | | | | | | | | |
|  | **Δ Dietary Approaches to Stop Hypertension (DASH)** | | | | | | |  | | **Δ Protein Diet score** | | | | | | | |  | |
|  | Decr/Maint | | **T1** | | **T2** | | **T3** |  | | **Decr/Maint** | | **T1** | | **T2** | | **T3** | |  | |
|  | n= 2793 | | n= 1131 | | n= 899 | | n= 852 | p-value | |  | |  | |  | |  | | p-value | |
| Baseline 17-item erMedDiet score, points | 9 ± 2.7 | | 8.4 ± 2.6 | | 8.1 ± 2.5 | | 7.4 ± 2.4 | <0.01 | | 8.7 ± 2.7 | | 8.4 ± 2.7 | | 8.2 ± 2.6 | | 7.9 ± 2.5 | | <0.01 | |
| Baseline Trichopoulou-MedDiet, points | 4.7 ± 1.6 | | 4.3 ± 1.6 | | 4.1 ± 1.6 | | 3.8 ± 1.6 | <0.01 | | 4.6 ± 1.6 | | 4.2 ± 1.6 | | 4.1 ± 1.6 | | 3.9 ± 1.6 | | <0.01 | |
| Baseline DASH, points | 26.2 ± 4.8 | | 23.5 ± 4.6 | | 22.0 ± 4.4 | | 19.3 ± 3.8 | <0.01 | | 24.6 ± 5.2 | | 23.5 ± 5.2 | | 22.9 ± 5.2 | | 22.2 ± 4.9 | | <0.01 | |
| Baseline Protein Diet, points | 10.5 ± 3 | | 10 ± 2.9 | | 9.5 ± 3 | | 9 ± 2.8 | <0.01 | | -2.3 ± 2.1 | | 1.5 ± 0.5 | | 3.5 ± 0.5 | | 6.3 ± 1.7 | | <0.01 | |
| Age, years | 65.3 ± 4.9 | | 64.8 ± 4.8 | | 64.8 ± 5 | | 64.7 ± 4.8 | <0.01 | | 65.2 ± 4.9 | | 65.1 ± 4.8 | | 64.8 ± 4.9 | | 64.7 ± 5 | | 0.12 | |
| Women, % (n) | 51.2 (1431) | | 47.9 (542) | | 47.4 (426) | | 37.8 (322) | <0.01 | | 48 (1566) | | 49.1 (591) | | 46.8 (319) | | 46.5 (245) | | 0.71 | |
| BMI, kg/m^2^ | 32.5 ± 3.5 | | 32.5 ± 3.4 | | 32.4 ± 3.3 | | 32.7 ± 3.5 | 0.35 | | 32.4 ± 3.4 | | 32.6 ± 3.5 | | 32.6 ± 3.5 | | 32.6 ± 3.4 | | 0.24 | |
| PA, METS/min/week | 2597.5 ± 2322.2 | | 2542 ± 2381.2 | | 2461.8 ± 2452.5 | | 2413.6 ± 2202.9 | 0.16 | | 2583.4 ± 2349.9 | | 2527.8 ± 2373.3 | | 2455.4 ± 2270.3 | | 2379.9 ± 2267.7 | | 0.21 | |
| Energy intake, kcal/d | 2355.9 ± 533.7 | | 2317.8 ± 563.5 | | 2385.9 ± 558.3 | | 2476.8 ± 559.8 | <0.01 | | 2364.8 ± 552 | | 2379.8 ± 542.1 | | 2390.9 ± 534.5 | | 2365.9 ± 571.8 | | 0.64 | |
| Protein intake, % energy | 16.9 ± 2.8 | | 16.7 ± 2.8 | | 16.6 ± 3 | | 16.4 ± 2.8 | <0.01 | | 17 ± 2.8 | | 16.5 ± 2.8 | | 16.4 ± 2.7 | | 15.9 ± 2.7 | | <0.01 | |
| Smoking status, % (n) |  | |  | |  | |  | 0.02 | |  | |  | |  | |  | | 0.40 | |
| Never smoked | 45.9 (1283) | | 44.5 (503) | | 43.2 (388) | | 40.6 (346) |  | | 44.0 (1436) | | 44.8 (540) | | 48.0 (327) | | 41.2 (217) | |  | |
| Former smoker | 41.2 (1150) | | 44.3 (501) | | 42.7 (384) | | 47.3 (403) |  | | 43.1 (1407) | | 42.8 (516) | | 40.4 (275) | | 45.5 (240) | |  | |
| Current smoker | 12.9 (360) | | 11.2 (127) | | 14.1 (127) | | 12.1 (103) |  | | 12.8 (419) | | 12.4 (540) | | 11.6 (327) | | 13.3 (70) | |  | |
| Education level, % (n) |  | |  | |  | |  | 0.02 | |  | |  | |  | |  | | 0.07 | |
| Primary education | 51.1 (1427) | | 48.4 (547) | | 46.9 (422) | | 49.4 (421) |  | | 50.7 (1654) | | 49.1 (591) | | 48 (327) | | 46.5 (245) | |  | |
| Secondary education | 27 (754) | | 29.1 (329) | | 33.0 (297) | | 30.6 (261) |  | | 27.3 (889) | | 30.4 (366) | | 31.3 (213) | | 32.8 (173) | |  | |
| Academic or graduate | 21.9 (612) | | 22.6 (255) | | 20.0 (180) | | 20 (170) |  | | 22 (719) | | 20.6 (248) | | 20.7 (141) | | 20.7 (109) | |  | |
| eGFR, mL/min/1.73m^2^ | 84.4 ± 13.8 | | 84.1 ± 13.8 | | 83.6 ± 14.5 | | 84.1 ± 14.2 | 0.46 | | 84.5 ± 13.7 | | 83.6 ± 13.9 | | 83.8 ± 14.4 | | 84.1 ± 14.9 | | 0.29 | |
| CKD, % (n) | 6.3 (176) | | 6.4 (72) | | 7.8 (70) | | 7 (60) | 0.43 | | 6.1 (197) | | 7.7 (93) | | 6.6 (45) | | 8.2 (43) | | 0.11 | |
| Type 2 diabetes, % (n) | 30.8 (859) | | 32.4 (366) | | 28.7 (258) | | 28.8 (245) | 0.21 | | 30.8 (1003) | | 30.8 (371) | | 30.3 (206) | | 28.1 (148) | | 0.66 | |
| Hypertension, % (n) | 83.2 (2324) | | 84.9 (960) | | 85 (764) | | 86 (733) | 0.17 | | 84.8 (2766) | | 83.7 (1009) | | 83.4 (568) | | 83.1 (438) | | 0.60 | |
| Hypercholesterolemia, % (n) | 70.1 (1959) | | 70.2 (794) | | 67.7 (609) | | 69.1 (589) | 0.55 | | 69.6 (2271) | | 69.6 (839) | | 69.8 (475) | | 69.5 (366) | | 1.00 | |
| Abbreviations: Decr/Maint, Decrease/Maintenance; MedDiet, Mediterranean Diet; BMI, Body Mass Index; PA, Physical activity; eGFR, estimated Glomerular Filtration Rate; CKD, Cronic Kidney Disease (eGFR<60 mL/min/1.73m^2^).  Values are presented as percentages (n) for categorical variables and means ± standard deviations (SD) for continuous variables. P-value was calculated by chi-square or one-way analysis of variance test for categorical and continuous variables, respectively. | | | | | | | | | | | | | | | | | | | |

**.**

| **Supplementary table 3. Baseline characteristics of excluded vs included individuals for analysis** | | | |
| --- | --- | --- | --- |
|  | PREDIMED-Plus participants | |  |
|  | Excluded | Included |  |
|  | n=4002 | n=5675 | p-value |
| Baseline 17-item erMedDiet score, points | 8.5 ± 2.7 | 8.5 ± 2.7 | 0.535 |
| Baseline Trichopoulou MedDiet, points | 4.3 ± 1.6 | 4.4 ± 1.6 | 0.512 |
| Baseline DASH, points | 23.8 ± 5.2 | 24.0 ± 5.2 | 0.271 |
| Age, years | 65.5 ± 5.1 | 65.0 ± 4.9 | <0.01 |
| Women, % (n) | 53.05 (2123) | 47.95 (2721) | <0.01 |
| BMI, kg/m^2^ | 32.9 ± 3.5 | 32.5 ± 3.4 | <0.01 |
| PA, METS/min/week | 2109.5 ± 2080.9 | 2537.3 ± 2338.3 | <0.01 |
| Energy intake, kcal/d | 2636.2 ± 906.1 | 2371.2 ± 549.6 | <0.01 |
| Protein intake, % energy | 16.7 ± 2.8 | 16.7 ± 2.8 | 0.849 |
| Smoking status, % (n) |  |  | <0.01 |
| Never smoked | 48.6 (1945) | 44.41 (2520) |  |
| Former smoker | 38.6 (1544) | 42.9 (2438) |  |
| Current smoker | 12.8 (513) | 12.6 (717) |  |
| Education level, % (n) |  |  | <0.01 |
| Primary education | 45.2 (540) | 49.6 (2817) |  |
| Secondary education | 28.9 (345) | 28.9 (1641) |  |
| Academic or graduate | 25.9 (309) | 21.4 (1217) |  |
| eGFR, mL/min/1.73m^2^ | 84.8 ± 14.3 | 84.2 ± 13.9 | 0.167 |
| CKD, % (n) | 5.7 (63) | 6.7 (378) | 0.229 |
| Type 2 diabetes, % (n) | 30.4 (365) | 30.5 (1728) | 0.996 |
| Hypertension, % (n) | 79.5 (3182) | 84.3 (4781) | <0.01 |
| Hypercholesterolemia, % (n) | 69.8 (2795) | 69.6 (3951) | 0.817 |
| Abbreviations: Decr/Maint, Decrease/Maintenance; T, tertile; MedDiet, Mediterranean Diet; BMI, Body Mass Index; PA, Physical activity; eGFR, estimated Glomerular Filtration Rate; CKD, Cronic Kidney Disease (eGFR<60 mL/min/1.73m^2^).  Values are presented as percentages (n) for categorical variables and means ± standard deviations for continuous variables. P-value was calculated by chi-square or one-way analysis of variance test for categorical and continuous variables, respectively. | | | |

| **Supplementary table 4. Multivariable adjusted β-coefficients and 95% CI for changes in eGFR (ml/min/1.73m^2^) and multivariable adjusted odd ratios and 95% CI for eGFR decline (>10%) across categories of changes to the Mediterranean Diet (17-item erMedDiet score and Trichopoulou) and DASH Diet adherence in participants without CKD (eGFR >60 ml/min/1.73m2) at baseline after 1 year of follow-up (n=5297)** | | | | | |
| --- | --- | --- | --- | --- | --- |
|  | **Δ Mediterranean Diet (17-item erMedDiet score)** | | | | |
|  | **Decr/Maint** | **T1** | **T2** | **T3** | **p for trend** |
|  | (n = 1041) | (n = 1797) | (n = 1123) | (n = 1336) |  |
| Δ 17-item erMedDiet score | -1.2 ± 1.4 | 2.1 ± 0.8 | 4.5 ± 0.5 | 7.5 ± 1.5 |  |
| **Δ eGFR, ml/min/1.73m^2,^**^a^ | -2.30 (-2.88 to -1.71) | -1.44 (-1.84 to -1.03) | -1.29 (-1.74 to -0.83) | -0.64 (-1.16 to -0.12) |  |
| Multivariable model (β-coefficients and 95% CI) | 0 (Ref.) | 0.86 (0.19 to 1.53) | 1.01 (0.24 to 1.78) | 1.65 (0.77 to 2.54) | <0.001 |
| **eGFR decline** >10%, % (n) | 19.2 (200) | 15.9 (286) | 15.4 (173) | 15.0 (200) |  |
| Multivariable model (OR and 95% CI) | 1 (Ref.) | 0.72 (0.58 to 0.88) | 0.67 (0.52 to 0.86) | 0.59 (0.44 to 0.79) | 0.001 |
|  | **Δ Mediterranean Diet (Trichopoulou)** | | | | |
|  | (n = 3184) | (n = 982) | (n = 637) | (n = 494) |  |
| Δ Trichopoulou-MedDiet | -1.2 ± 1.2 | 1 ± 0 | 2 ± 0 | 3.5 ± 0.7 |  |
| **Δ eGFR, ml/min/1.73m^2,^**^a^ | -1.49 (-1.80 to-1.19) | -1.03 (-1.54 to -0.52) | -1.42 (-2.11 to -0.73) | -1.22 (-2.00 to -0.44) |  |
| Multivariable model (β-coefficients and 95% CI) | 0 (Ref.) | 0.46 (-0.14 to 1.07) | 0.07 (-0.70 to 0.84) | 0.27 (-0.60 to 1.15) | 0.386 |
| **eGFR decline** >10%**,** % (n) | 16.1 (514) | 16.7 (164) | 15.5 (99) | 16.6 (82) |  |
| Model 3 (OR and 95% CI) | 1 (Ref.) | 1.06 (0.86 to 1.29) | 0.99 (0.77 to 1.28) | 1.10 (0.82 to 1.48) | 0.605 |
|  | **Δ Dietary Approaches to Stop Hypertension (DASH)** | | | | |
|  | (n = 2617) | (n = 1059) | (n = 829) | (n = 792) |  |
| Δ DASH | -3.7 ± 3.3 | 2.0 ± 0.8 | 4.9 ± 0.8 | 9.5 ± 2.6 |  |
| **Δ eGFR, ml/min/1.73m^2^**^,a^ | -1.37 (-1.72 to -1.01) | -1.12 (-1.58 to -0.65) | -1.34 (-1.94 to -0.74) | -1.78 (-2.44 to -1.13) |  |
| Multivariable model (β-coefficients and 95% CI) | 0 (Ref.) | 0.25 ( -0.35 to 0.85) | 0.03 (-0.70 to 0.76) | -0.42(-1.22 to 0.39) | 0.520 |
| **eGFR decline** >10%**,** % (n) | 16.5 (432) | 14.7 (156) | 17.25 (143) | 16.2 (128) |  |
| Multivariable model (OR and 95% CI) | 1 (Ref.) | 0.89 (0.72 to 1.09) | 1.10 (0.87 to 1.39) | 1.04 (0.80 to 1.36) | 0.702 |
| Abbreviations: Decr/Maint, Decrease/Maintenance; T, tertile; eGFR, Estimated glomerular filtration rate; MedDiet, Mediterranean Diet; DASH, Dietary Approaches to Stop Hypertension.  ^a^Multivariable adjusted mean changes in eGFR (ml/min/1.73m^2^) after 1 year of follow-up.  Linear regression models were used to assess changes in eGFR by categories of changes in dietary patterns score. Logistic regression models were used to assess eGFR decline (>10%) by categories of dietary patterns score changes. Multivariable model was adjusted for baseline eGFR (except for logistic regression model), sex, age. BMI, smoking habits (never, current or former smoker), educational level (primary, secondary education, graduate), leisure time physical activity (METS/min/week), diabetes prevalence (yes/no), hypertension prevalence (yes/no), hypercholesterolemia prevalence (yes/no), center (categorized into quartiles by number of participants), intervention group, energy intake (kcal/day), each baseline dietary pattern score and 1-year changes in body weight.  *p-value < 0.05 | | | | | |
